# In vitro and in vivo toxicity of carbon dots with different chemical compositions

**DOI:** 10.1186/s11671-023-03891-9

**Published:** 2023-09-08

**Authors:** Halyna Kuznietsova, Alain Géloën, Nataliia Dziubenko, Alexander Zaderko, Sergei Alekseev, Vladimir Lysenko, Valeriy Skryshevsky

**Affiliations:** 1 Institute of High Technologies, Taras Shevchenko National University of Kyiv, Volodymyrska Street, 64, Kiev, 01601 Ukraine; 2 Corporation Science Park, Taras Shevchenko University of Kyiv, 60 Volodymyrska Str., Kiev, 01033 Ukraine; 3 Laboratoire Ecologie Microbienne (LEM), UMR CNRS 5557, INRAE 1418, VetAgroSup, Université Lyon 1, Domaine Scientifique de La Doua, 69100 Villeurbanne, France; 4 Department of Ecology, Faculty of Humanities and Natural Science, University of Presov, 17Th November Str. 1, 08001 Presov, Slovak Republic; 5 Faculty of Chemistry, Taras Shevchenko National University of Kyiv, Lva Tolstoho Street, 12, Kiev, 01033 Ukraine; 6 Light Matter Institute, UMR-5306, Claude Bernard University of Lyon/CNRS, Université de Lyon, 69622 Villeurbanne Cedex, France

**Keywords:** Carbon dots; ethylenediamine, Carboxyl, Phenol, Trifluoromethyl, Aniline, Toluidine remains, A549 cells, C57Bl6 mice

## Abstract

Carbon dots (CDs) are easy-obtained nanoparticles with wide range of biological activity; however, their toxicity after prolonged exposure is poorly investigated. So, in vitro and in vivo toxicity of CDs with the surfaces enriched with hydroxylated hydrocarbon chains and methylene groups (CD_GE), carboxyl and phenol groups accompanied with nitrogen (CD_3011), trifluoromethyl (CDF19) or toluidine and aniline groups (CDN19) were aimed to be discovered. CDs’ in vitro toxicity was assessed on A549 cells (real-time cell analysis of impedance, fluorescence microscopy) after 24 h of incubation, and we observed no changes in cell viability and morphology. CDs’ in vivo toxicity was assessed on C57Bl6 mice after multiple dosages (5 mg/kg subcutaneously) for 14 days. Lethality (up to 50%) was observed in CDN19 and CD_3011 groups on different days of dosing, accompanied by toxicity signs in case of CD_3011. There were no changes in serum biochemical parameters except Urea (increased in CDF19 and CD_3011 groups), nor substantial kidney, liver, and spleen injuries. The most impactful for all organs were also CD_3011 and CDF19, causing renal tubule injury and liver blood supply violation. Thus, CDs with a surface enriched with oxygen- and nitrogen-containing functional groups might be toxic after multiple everyday dosing, without, however, significant damages of internal organs in survived animals.

**Supplementary Information:**

The online version contains supplementary material available at 10.1186/s11671-023-03891-9.

## Introduction

During the last years, nanoscience has developed very extensively. Among a wide range of possible applications of nanoscience’s achievements, medical application of those occupies the leading position. Nanoparticles of very different origin and composition are extensively studied and are already in use for drug delivery and radiosensitization [1]. In recent years, attention is paid to carbon-based nanoparticles because of their excellent biocompatibility, ease of synthesis and unique physico-chemical properties. Carbon-based NPs are widely discovered, passing the preclinical and clinical trials (NCT04482803; NCT05229874; NCT04759820 [2]; NCT02724176 [3] and are extensively studied as drug carriers [4, 5] and therapeutics itself [6]. Furthermore, nanoparticles doped with metals are served as excellent visualization and therapeutics tools because of combining nanosize and metal properties [7, 8].

Carbon dots (CDs) are a novel type of carbon-based nanoparticles that possess unique characteristics. They are nearly spherical nanomaterials with a particle size smaller than 10 nm. CDs exhibit zero-dimensional spherical structures and possess various properties such as water solubility, conductivity, optical properties, low toxicity, and biocompatibility. These properties make them highly promising for applications in drug delivery and diagnostics [4]. Additionally, when exposed to light energy, CDs can generate reactive oxygen species, making them suitable for use in photodynamic therapy [9].

CDs could be bioactive not only because of irradiation, but of their own physico-chemical properties, like size, surface chemical composition and charge. Being able to be doped with different heteroatoms, CDs could realize fluorescent and luminescent properties which vary depending on the origin of the heteroatom [10] and allow them to be used in bioimaging. Additionally, CDs could be doped with rare earth metals like Gadolinium and served as contrasting agents in MRI [9], while their ability to prevent metal toxicity with preserving and/or improving its contrasting properties strongly depends on their chemical composition [11]. Furthermore, to be used as therapeutics, they should be relatively safe. Despite the main current purpose of such molecules as MRI-contrasting agents is their serve as a diagnostic tool, the possibility of their application as therapeutics is on air. Therefore, to be candidates as anticancer drugs, these substances should be relatively safe after prolonged exposure. Thus, the purpose of this work was to discover in vitro and in vivo toxicity of CDs with different chemical compositions in order to assess their availability as potential agents for multi-dosing therapy.

## Materials and methods

### CDs synthesis and characterization

#### Ethylenediamine CD (CD_GE)

Briefly, CD_GE CDs were synthesized as follows: glucose monohydrate (0.495 g, 2.5 mmol) and ethylenediamine (170 µL, 2.5 mmol) (Table 1) were dissolved in 5 mL of water and sealed in a teflon-lined stainless-steel autoclave. The autoclave was heated to 180 °C with 5 °C/min heating rate, kept for 3 h at this temperature and naturally cooled. Resulted solution was evaporated at 60 °C in a rotary evaporator and additionally dried at 60 °C under 10^–3^ bar vacuum. The solid was dissolved in water (1 mg/mL) under sonication. Resulted transparent dark-brown solution was centrifuged at 14,500*g* for 10 min, and negligibly small precipitate was disposed. Detailed description of the synthesis and characterization, as well as fraction distribution, is provided at [12].

**Table 1:** Reagents required for CDs synthesis

Reagents, mmol	CD_GE	CD_3011	CDN19	CDF19
Glucose Monohydrate	2.5			
Ethylenediamine	2.5			
Urea		167	167	167
Anhydrous citric acid		83	83	83
3-(Trifluoromethyl)aniline			21	
*m*-Toluidine				21

The structure of CD_GE was confirmed by Fourier transform infrared spectroscopy (FTIR) (Nicolet Nexus 470 with Diamond Smart Orbit ATR accessory) and proton nuclear magnetic resonance spectroscopy (^1^H NMR) (Agilent ProPulse 600 instrument, sample dissolved in D_2_O), CDs fraction distribution was assessed by ultraviolet–visible (UV–Vis) (Thermo Evolution 600) and photoluminescence (PL) spectroscopies (Shimadzu RF-6000).

#### CD_3011, CDN19, CDF19 CDs

The synthesis of CD_3011, CDN19, CDF19 CDs was generally similar to the procedure for the synthesis of F-O-N-containing CDs described in [13]. Briefly, to prepare CD_3011, CDN19 and CDF19, we took a three Pyrex glass bottles, and in every bottle 10 g of urea (167 mmol, ACS reagent, 99.0–100.5%, Sigma-Aldrich), 16 g of anhydrous citric acid (83 mmol, ACS reagent, ≥ 99.5%), and in second and third case, 21 mmol of corresponding amine (3.38 g of 3-(Trifluoromethyl)aniline, ≥ 99%, Sigma-Aldrich, or 2.25 g of *m*-Toluidine, 99%, Sigma-Aldrich) were placed (Table 1). Mixtures were homogenized and placed in the oven at 135 °C for 3 h. During this stage, synthesis ammonia and other gases and vapors were evolved. Then, temperature was increased to 165 °C for 2 h. Obtained products (as shining brittle solidified melt with numerous bubbles) were dissolved in 135 mL of solution consisting of water: ethanol: ammonia = 100: 10: 25, the residues were filtered off. Then, approx. 30 mL of concentrated hydrochloric acid was added and left overnight. Precipitate was dried on air at 120 °C.

The structure of CD_3011, CDN19, CDF19 was discovered by thermogravimetry and differential scanning calorimetry (NETZSCH STA 449 F3 Jupiter®—Thermal Analysis System, Germany).

### Cell toxicity of carbon dots

As tested CDs were developed as potential theranostic agents against cancer, human lung cancer A549 cells (obtained from Claude Bernard University of Lyon) were used to test CDs toxicity as a preliminary testing in order to determine could these CDs be applied in vivo. This cell line was chosen because of its applicability to CDs testing [14]. Cells were cultured in DMEM (1 g/L glucose) with 10% FBS, 100 U/mL of penicillin, and 100 μg/mL of streptomycin (PAA Laboratories, Les Mureaux, France) at 37 °C and 5% CO_2_.

#### Cytotoxicity

Cell number measurements were conducted using the Real-Time Cell Analysis of Impedance (xCELLigence, RTCA, Agilent, Santa Clara, USA) technique. The cells were cultivated on a specialized 96-well plate equipped with electrodes at the bottom. This system gauges the electrical impedance by analyzing the interdigitated microelectrodes positioned in the culture wells. The measurements involved applying an alternating excitation signal with a control voltage amplitude of 20 mV at three distinct frequencies (10, 25, and 50 kHz) through the microelectrodes of the E-plate. The voltage drop across the electrodes was monitored, and by calculating the quotient of voltage and current, the impedance was determined. The software generated the cell index by processing the impedance data over time. The cell index corresponds to the cell number, single cell surface area, and adhesion factor. In basal conditions of a specific cell line, the cell number has the greatest impact on the cell index. Each curve represents the average cell index obtained from measurements conducted in four wells. The cells were cultivated for 48 h until the cell index reached approximately one, indicating the linear proliferative phase. Subsequently, the culture medium was replaced with a fresh medium containing CDs at various concentrations: 0 mg/mL (control), 0.25 mg/mL, 0.5 mg/mL, 0.75 mg/mL, 1.0 mg/mL, 1.5 mg/mL, and 2.0 mg/mL [15]. The impedance was recorded every 15 min in a standard 37 °C cell culture incubator with 5% CO_2_. After 24 h, the medium was discarded and replaced with fresh medium, and measurements were continued for an additional 12 h following the washing of cells.

#### Fluorescence assay

Cells were plated using Scepter counter^®^ (Millipore, USA) at 2500 cells/well in 96 well plate. After 48 h, 100 µL of culture medium containing CDs solutions at the concentrations used in cytotoxicity assay were added to the wells. Cells were incubated in presence of CDs for 24 h. Cell imaging was performed on Cytation 3 platform: Images were taken at × 4 to × 40 magnification using Cytation 3 cell imaging reader (Biotek Instrument Inc., Colmar, France) on living cells maintained at 37 °C. Fluorescence intensities were measured at magnification × 20 with filters 469/525 (green), 586/647 (red), 377/447 (blue), 8 wells per CD were analyzed. Image and fluorescence data were retrieved using the software Gen5 2.08 (Biotek Instruments, Winooski, USA) [16].

### *Toxicity of carbon dots *in vivo

#### Animals

C57BL6 male mice 11–12 weeks old with initial body weight 19 ± 3 g were used in the study. Animals were kept in animal facility of Taras Shevchenko National University of Kyiv in standard conditions (12 h light/dark cycle, 50% humidity at 20–22 °C) and free access to standardized rodent diet and tap water.

#### Bioethics

All experiments with the animals were conducted in compliance with bioethics principles, legislative norms and provisions of the European Convention for the Protection of Vertebrate Animals used for Experimental and Other Scientific Purposes (Strasbourg, 1986), General Ethical Principles for Experiments on Animals, adopted by the First National Bioethics Congress (Kyiv, 2001), and ARRIVE and Animal Care guidelines. Protocol of the study was approved by Taras Shevchenko National University of Kyiv Animal Care and Use Committee (protocol #9 dated Nov 10 2021).

#### Design of the study

Mice were randomly assigned onto 5 treatment groups (*n* = 7 in each) and received CDN19, CD_3011, CDF19 and CD_GE solutions in NaHCO_3_ buffer 4.2% or a Vehicle (NaHCO_3_ buffer 4.2%, Yuria-Pharm, Ukraine) served as a control, subcutaneously (back neck region) at the volume of 5 mL/kg (which corresponds to CDs dose 5 mg/kg) daily during 14 consecutive days. The route of administration was chosen in order to serve that in the following efficacy study of CDs anticancer activity with allografted tumors (unpublished data). The dose of administration was chosen based on preliminary dose range finding study (unpublished data) and on the solubility of the substances. The size of the CDs in the buffer phase exceeded what is convenient for true solutions, making it more like a super-thin suspension. However, despite this, the phase appeared visually clear and transparent with no residue and maintained these properties for at least 6 months. So, we decided to refer to the CDs in the buffer as a solution and used that terminology here and below.

All the substances were prepared once in a sterile condition and kept at + 4 °C. Sterile condition was maintained during the substance sack for each administration. At the 15th day, the study mice were anesthetized by 2,2,2-tribromoethanol (150 mg/kg) (Sigma-Aldrich, USA) and sacrificed by cervical dislocation.

#### Examinations and observations

The overall wellbeing and weight of the mice were monitored on a daily basis. The condition of their skin, fur, eyes, mucous membranes, respiratory system, posture, and changes in spontaneous activity was assessed. A comprehensive scoring system, outlined in Table 2, was used for evaluation. These observations were conducted right after the initial administration and once daily throughout the observation period.

**Table 2:** Gross toxicity scale [17, 18]

Sign	Expression, score^a^				
	0	1	2	3	4
General appearance	Normal		Unnatural posture/hunched pose	Emaciation	
Hypo/hyperkinesia	Absent	Decrease/increase activity	Drowsiness/aggression	Unresponsive to extraneous activity and provocation	
Movement activity	Normal	Dysbasia/circling	tremor	Convulsions, limb paralysis	
Respiration alterations	Absent	Deep/heavy/rapid/shallow			Respiratory arrest
Skin/coat injuries	Absent	Redness	Wounds	Abscess	Necrosis
Piloerection	Absent	Present			
Eyes conditions alterations	Absent	Pale/clouded/ tearing	Sunken/inflamed/half-closed	Closed eyes, do not open on touch	
Exudation	Absent		ptyalism/nasal Exudation		
Defecation changes	Absent	Abdomen abnormally enlarged/ loose stool	Constipation	Diarrhea	Defecation with blood
Oedema/ alterations including the site of administration	Absent	Oedema/other changes at the site of administration	Not at the site of administration		
Body temperature	Normal		Increased/decreased		
Vocalization	Absent	Occasional	Consistent		

^a^Death of animal is considered to be equal 10

#### Biochemical assays.

After the mice were sacrificed, blood samples for biochemical analysis were obtained from the orbital sinuses. The collected blood was allowed to sit for 20–60 min to allow for the formation of a fibrin clot. Subsequently, the samples were centrifuged at 5400*g* for 20 min at a temperature of + 4 °C. The resulting blood serum was collected and stored at − 20 °C until further analysis. The levels of alanine aminotransferase (ALT), aspartate aminotransferase (AST), gamma-glutamyl transferase (GGT), lactate dehydrogenase (LDH), urea, and creatinine were determined using a fully automated chemistry analyzer (MF-240, MedFuture LLC, USA) and standard reagent kits (Cormay, Poland) as per the manufacturer's protocols.

#### Histological assays

Following the sacrifice of the animals, liver (left lateral lobe), kidney, and spleen samples were promptly collected. The number of animals used for each treatment group was *n* = 3 for the CDN19- and CD_3011-treated groups, and *n* = 7 for the CDF19-, CD_GE-, and Vehicle-treated groups. The samples were fixed in 10% neutral buffered formalin (Ukrorgsynthesis Ltd, Ukraine) for a duration of 7 days. After formalin fixation, the samples underwent dehydration using ethanol solutions (Ukrzoovetprompostach, Ukraine) and were embedded in paraffin type 6 (Thermo Scientific, USA). The paraffin blocks were then cut into 5 µm thick sections, which were deparaffinized and stained with hematoxylin and eosin (H&E, Sigma-Aldrich, USA) using standard techniques [19]. The stained sections were examined under a light microscope by a pathologist who was blinded to the treatment groups. A minimum of 5 random fields of view at × 100 and × 400 magnifications were analyzed from at least 3 sections. The pathological features were assessed in a semi-quantitative manner, and the specific scoring systems are outlined in Table 3.

**Table 3:** Histopathology signs [18]

	Kidney
Glomerulus state	Glomerulus shrinkage/capsule space dilation
Tubular state	Epithelial cell flattening, epithelial cell vacuolation, epithelial cell desquamation/loss, loss of brush border, tubular atrophy, tubular dilation
Inflammation/necrosis signs	Eosinophilic cast deposition, tubular epithelial necrosis^a^, interstitial nephritis, glomerulonephritis, hemorrhages^a^, vessel dilation
Abnormal regeneration	Connective tissue accumulation, tubular hyperplasia, tubular basophilia

	Liver
Hepatocytes alteration	Lipid dystrophy, ground-glass hepatocytes, eosinophilic alteration, basophilic alteration
Regeneration	Hepatocellular hypertrophy, polyploid cells
Inflammation/necrosis signs	Vessel congestion/dilation, blood sinusoids dilation, lympho-histiocytes accumulation loci, Kupffer cell diffuse accumulation, necrotic loci^a^, apoptotic loci^a^
Connective tissue accumulation	

	Spleen
Hypo/hyperplasia	Lymphoid hypoplasia as reduced white pulp, lymphoid atrophy, red pulp hyperplasia, white pulp hyperplasia, megakaryocytosis, marginal zone hyperplasia
Inflammation/necrosis signs	Necrosis^a^, hemorrhages^a^
Accumulations	Lipid accumulation, pigmentation, connective tissue accumulation

Trait intensity score: “0”—not observed or less than 10%; “+”—less than 50%; “++ ”—less than 80%; “+ + + ”—more than 80% of field of view/number if counted

^a^“0”—not observed; “+”—small occasional, “++”—small frequent, “+++”—large occasional, “++++”—large frequent

### Statistical analyses

Homogeneity of variance was assessed using the Levene test. Statistical analysis of the data was performed using one-way analysis of variance (ANOVA) with the Tukey post hoc test. Mann–Whitney *U*-test for independent samples was used for the analysis of clinical signs scores. Log-rank Mantel–Cox test with further Kaplan-Meier plotting was used to assess animal survival. The difference was considered statistically significant at *p* < 0.05.

## Results

### CDs characterization

**Fig. 1:**
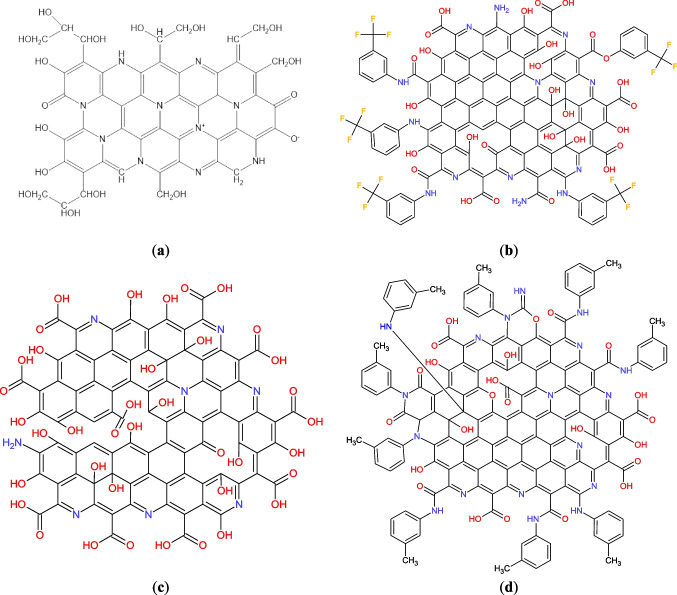
Suggested structures of CD_GE (**a**), CDF19 (**b**), CD_3011 (**c**) and CDN19 (**d**) samples

The proposed structures of the CDs are presented in Fig. 1. In general, every CD is a largely amorphous carbon core with the presence of graphene-like areas and contains different amounts of functional groups.

**Fig. 2:**
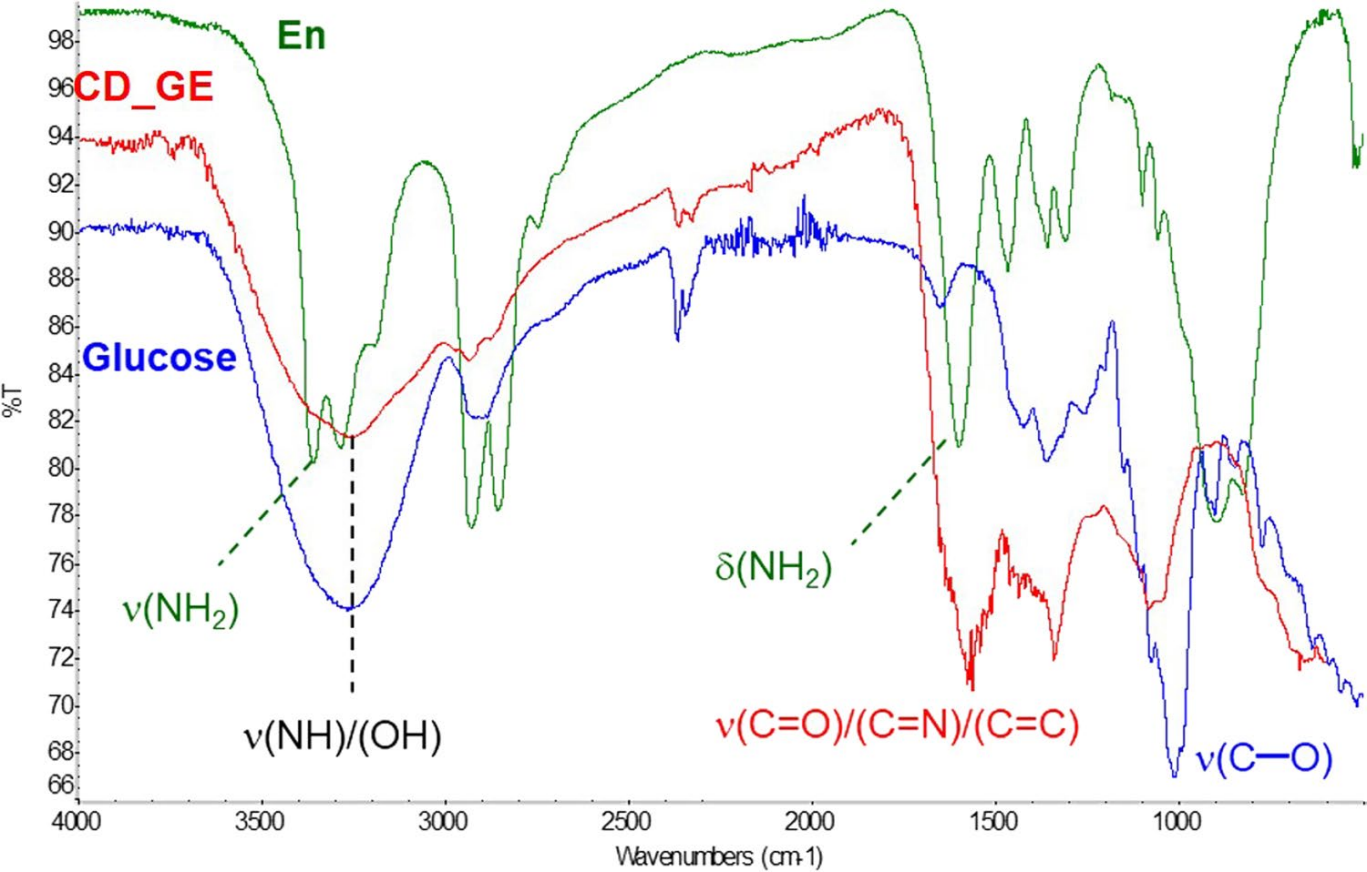
FTIR spectra of ethylenediamine (dark-green), glucose (blue) and CD_GE (red)

CD_GE contains a significant amount of O–H and N–H functionalities (as evidenced by wide band at 3265 cm^−1^), ensuring its hydrophilicity and high solubility in water. Intense FTIR bands at 1575 cm^−1^ (conjugated C=C and C=N double bonds) and 1070 cm^−1^ (single C–O bonds) absent in the spectra of initial compounds, confirmed the cross-coupling of glucose and ethylenediamine via Maillard reaction and formation of CD_GE (Fig. 2). The most intense signals in ^1^H NMR spectrum (Additional file 1: Fig. S1) could be related to –HC(OH)– protons of hydroxylated hydrocarbon chains of cross-linked glucose molecules (2.5–4 ppm), and methylene groups (1.2–2.2 ppm) correspond to the main structural fragments of CD_GE shown on Fig. 1a.

**Fig. 3:**
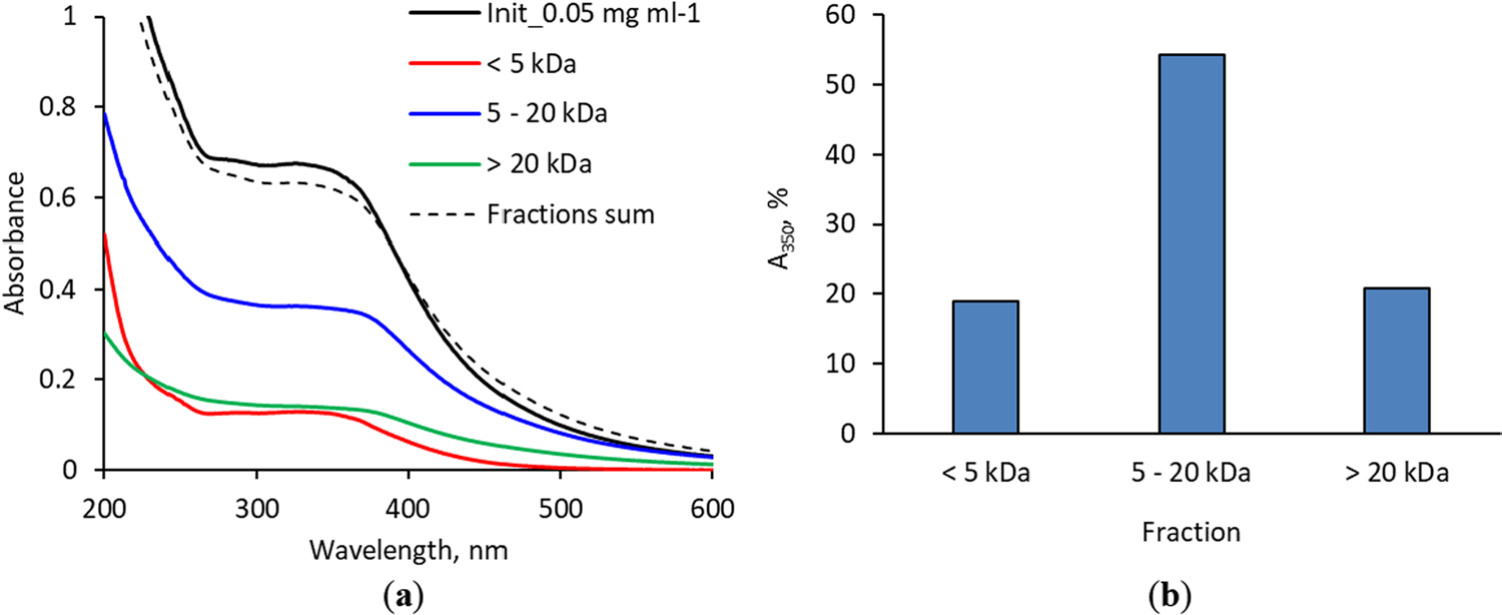
UV–Vis spectra of initial CD_GE solution and its fractions (the solutions were 20-fold diluted for spectra recording) (**a**); the fraction distribution of the CD_GE, calculated from optical absorbance at 350 nm (**b**)

CD_GE size separation was performed by passing its aqueous solution (1 mg/mL concentration) through 20,000 and 5000 MWCO Pall Microsep™ centrifugal filters and collecting the fractions > 20 kDa, 5–20 kDa and < 5 kDa. CD_GE faintly retained on the membranes after fraction collection, so quantitative separation can be assumed. UV–Vis spectra of initial solution, fractions after separation and sum of the fractions’ spectra are shown on Fig. 3. All the spectra demonstrated the absorbance decrease as the wavelength increased, with a wide shoulder near 350 nm. Comparing the absorbance intensity at 350 nm allowed rough estimation of particle size distribution: the major fraction possesses the MW between 5 and 20 kDa. These data were also confirmed by PL spectrum (Additional file 1: Fig. S2).

**Fig. 4:**
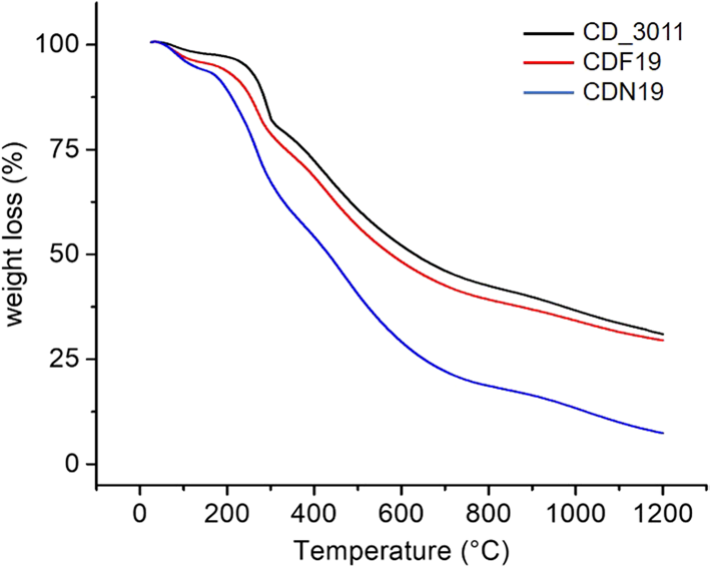
Thermogravimetry data for CD_3011, CDN19 and CDF19

CD_3011, CDN19 and CDF19 sample characterization was performed by thermogravimetric instrument NETZSCH STA 449F3 with using alumina crucible and heating rate of 10 °C/min. Purged argon gas was used for protective atmosphere with flow rate 50 mL/min. At a given heating rate in the temperature range from room temperature to 150 °C (Δ*w*1), water is physically desorbed, as well as alcohol residues and other low molecular weight products (temperature of the peak of desorption at 82–85 °C) from the surface of the carbon material. In the temperature range of 150–365 °C (Δ*w*2), carbon materials are characterized by weight loss due to dehydration reactions, mainly because of the interaction of surface carboxyl groups with each other, and with phenolic and amine groups, with the formation of anhydride, lactone and amide groups, respectively. The highest weight loss for CDN19 sample could be explained by a large amount of nitrogen in the sample. In the next temperature range of 365–830 °C, the main processes leading to weight loss (Δ*w*3) are the decarboxylation of carboxyl groups with CO2 release (at the temperatures 365–500 °C), and decomposition of phenolic groups accompanied by CO and CO_2_ release (at the temperatures of 500–830 °C). In the temperature range of 830–1200 °C (Δ*w*4), CO desorption occurs mainly due to the decomposition of ether, carbonyl, and especially quinone groups. The exact values of weight loss for each temperature range are provided in Table 4 and Fig. 4. The tested samples are characterized by very large total weight losses (Δ*w*) of 68.8%, 70.5% and 92.4%, in the series CD_3011—CDF19—CDN19, which could indicate that these samples contain a very large number of functional groups, especially CDN19 one. Differential TG data are represented on Additional file 1: Fig. S3 and Table S1 and confirmed by differential scanning calorimetry (Additional file 1: Fig. S4). CD_3011, CDN19 and CDF19 UV–Vis absorption and PL spectra can be found in [11].

**Table 4:** Thermogravimetry data for CD_3011, CDN19 and CDF19

Sample	r.t.–150	Temperature range (°C)			
		150–365	365–830	830–1200	r.t.–1200
		Weight loss (%)			
	Δ*w*1	Δ*w*2	Δ*w*3	Δ*w*4	Δ*w*
CD_3011	2.3	21.5	34.4	10.6	68.8
CDF19	4.4	23.6	33.6	8.9	70.5
CDN19	6.4	35.5	40.0	10.5	92.4

Thus, CD_3011 particle contains a large number of oxygen-containing groups, represented by groups of carboxyl and phenol types, and some nitrogen in the matrix. Carboxyl type groups provide acidic properties of nanoparticles; otherwise, nitrogen provides basicity. The total content of oxygen-containing groups is 68.8% by weight. Some properties and composition of such particles were previously investigated in [20].

CDF19 contains numerous oxygen-containing groups, predominantly represented by groups of carboxyl and phenol types. The total weight loss conditioned, mainly by decomposition of oxygen-containing groups, is up to 70.5% by weight. A distinctive feature of this CD is the presence of fluorine (total fluorine content is 1.35 mmol/g) as trifluoromethyl groups that provide some hydrophobic centers on the nanoparticle surface. At the temperatures above 350 °C, fluorine is eliminated from carbon materials as hydrogen fluoride because of pyrohydrolysis [21]. However, no additional signals that could be unambiguously attributed to this process were observed due to the high content of oxygen functional groups in the sample.

The CDN19 nanoparticle contains a lot of oxygen-containing groups, represented by carboxyl and phenol ones. The total weight loss caused by oxygen-containing groups and aniline remains is 92.4% by weight (68.8%, for CD sample), so we can suggest a presence of 23.6% of toluidine remains. A distinctive feature of this type of CD is the presence of very large amount of different surface functionalities including oxygen, nitrogen groups and aniline remains [22–24].

### In vitro* toxicity*

#### Cytotoxicity assay

**Fig. 5:**
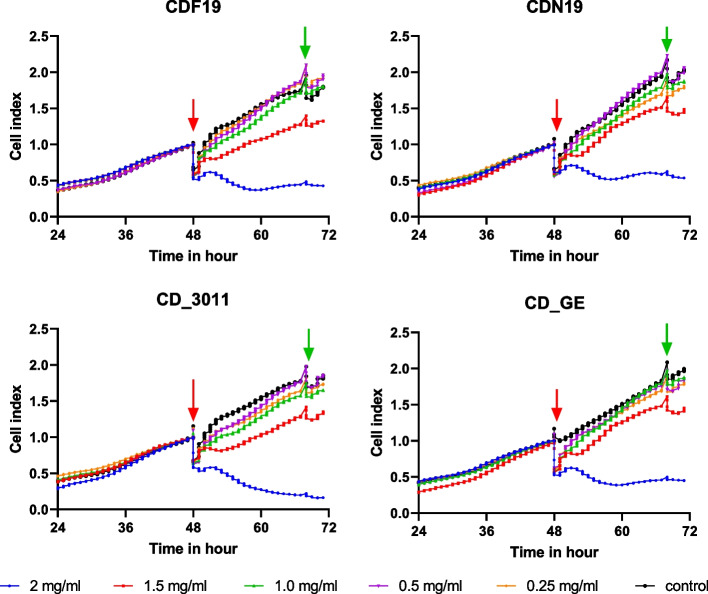
Cell index curves for A549 cells exposed to different CDs during 24 h. Arrows show the times of addition (red) and washing out (green) of the CDs. Each curve is a mean of the curves recorded from 4 wells

According to cell index measurement, all the CDs revealed no toxicity against cells if applied at concentration 1.5 mg/mL and lower. If applied at the highest concentration (2.0 mg/mL), CDF19, CDN19 and CD_GE demonstrated cytostatic activity (cell growth inhibiting), CD_3011—probably cytotoxic activity (Fig. 5). It should be noted that, however, then, even the concentration 1.0 mg/mL is high, and no toxicity under this concentration in in vitro assay could be considered as proof of the substance’s safety at least at acute exposure.

#### Fluorescence assay

**Fig. 6:**
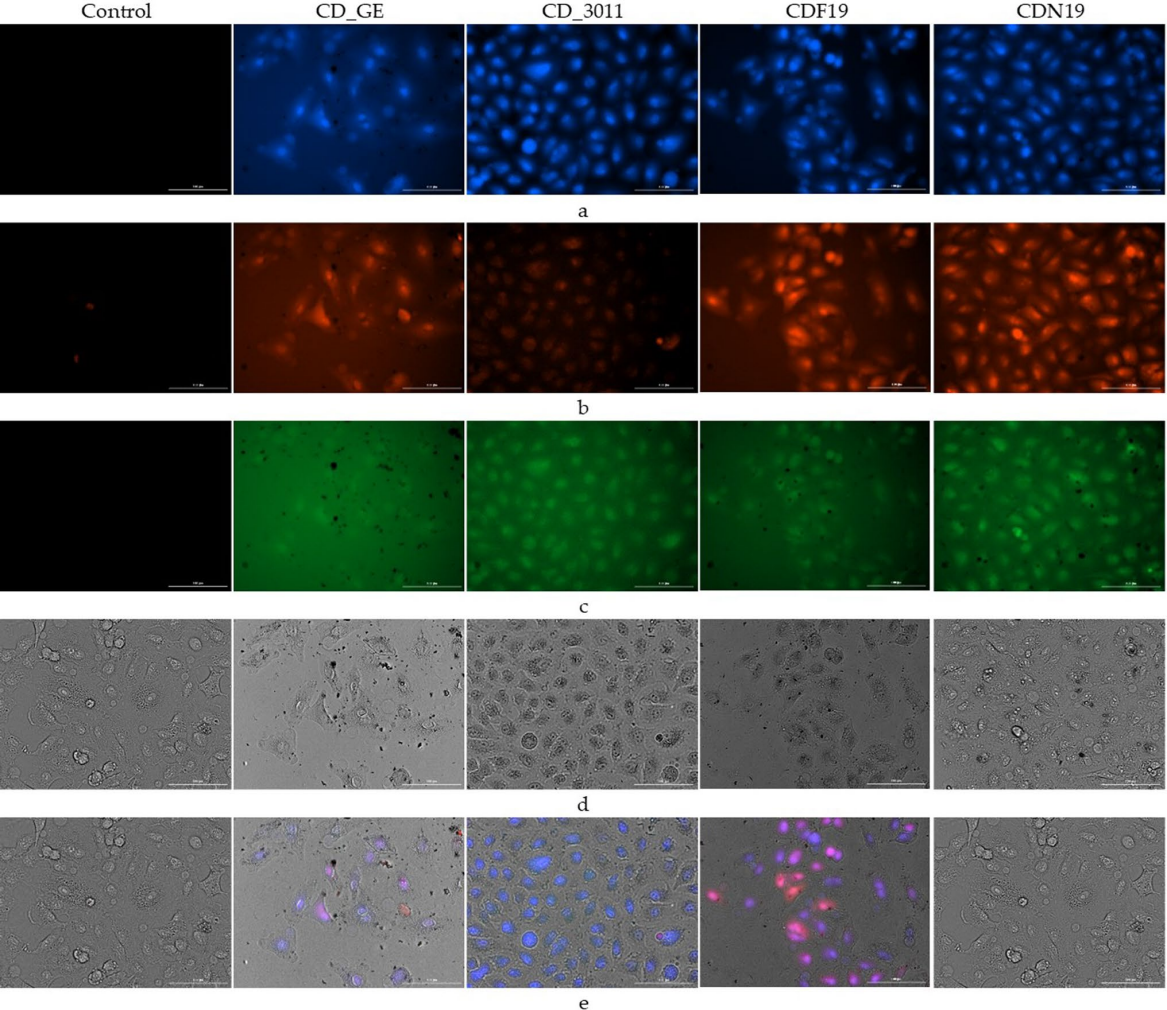
Representative fluorescent and optical microscopic images of live A549 cells exposed during 24 h to different CDs at 1 mg/mL using Cytation3. Eight wells were analyzed for each CD and control. Excitation and emission to detect the fluorescence of CDs were as follows: (**a**) blue 377/447; (**b**) red 469/525; (**c**) green 586/647; visible light (**d**); and merge (**e**). Scale white bar represents 100 µm

According to the microscopic analysis of cells incubated with CDs during 24 h at a concentration 1 mg/mL (as that revealed no cytostatic/cytotoxic activity), no substantial change in cellular morphology was observed, suggesting an absence of toxicity, despite the CDs association with the cells attested by the fluorescence (confirmation of the CDs fluorescence in used diapason see in Supplementary Figure S2 and [13]). No fluorescence was observed in control cells not exposed to CDs. Blue, red and green fluorescence were observed in cells exposed to CD_GE, CDF19 and CDN19. Red fluorescence was weak in cells exposed to CD_3011 (Fig. 6). As wavelength of fluorescence peak depends on the CDs size [25], we might suggest that CDs with the size not exceeded 20 kDa were predominantly accumulated into the cells. However, detailed determination of CDs accumulation within the different cellular compartment or even assertion about their accumulation inside the cells and not on their surface was not possible with used technique and require more thorough investigations to be confirmed. Then, cell shapes were not affected following the incubation in presence of CDs at 1 mg/mL, arguing for a lack of toxicity until that concentration. This is in good agreement with the literature data [26] and confirms the low toxicity of these CDs at least after acute exposure.

### In vivo* toxicity*

#### Gross toxicity findings

**Fig. 7:**
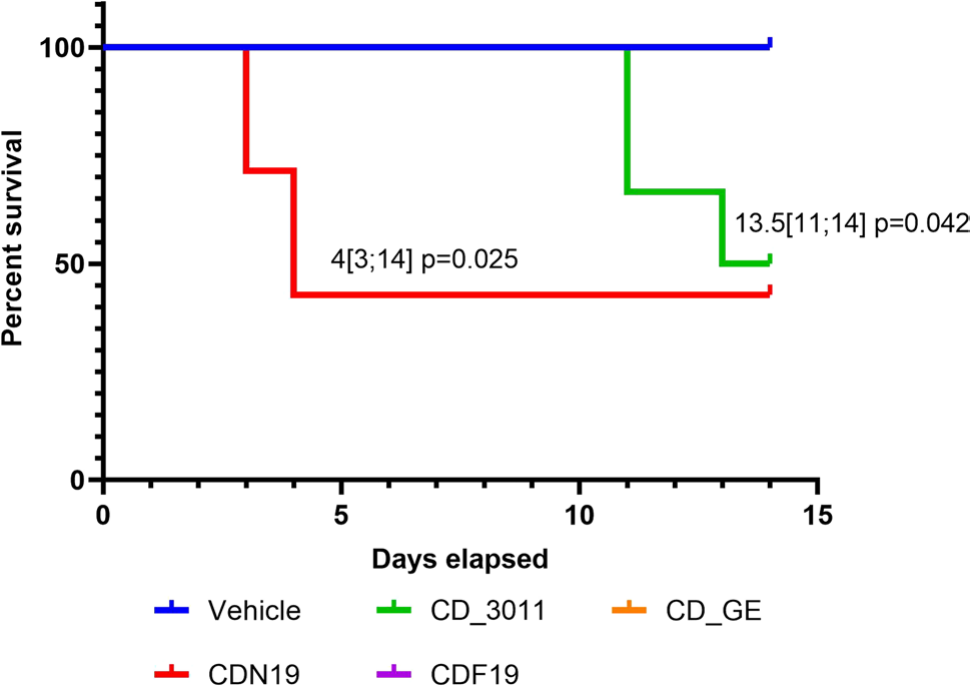
Kaplan–Meier survival plots for mice treated with CDN19 and CD_3011 (the groups where mortality was observed) compared to Vehicle-treated ones served as a control. Medians survival with respective p values are provided

Lethality was observed in CDN19- and CD_3011-treated groups only (4 out of 7 mice on the 4–5 days, and 3 out of 6 mice on the 12–14 days, respectively, Fig. 7). Moreover, animals’ death in CDN19-treated group was not accompanied by gross toxicity signs, whereas in CD_3011-treated animals, decreased activity and hunched posture were observed during 2 days before death. Application of CDF19 and CD_GE probably did not affect mice wellbeing during the study period.

**Fig. 8:**
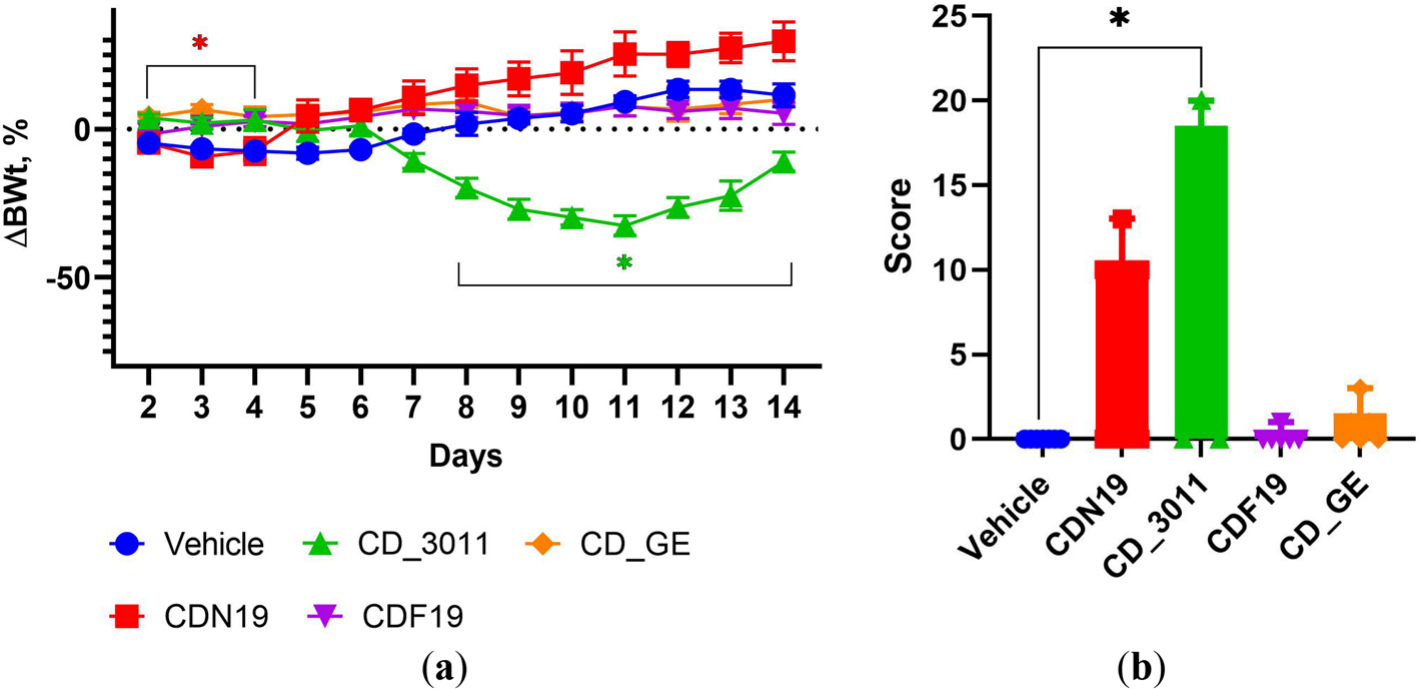
Body weight dynamics (**a**) and terminal toxicity scores (**b**) of mice treated with CDN19, CD_3011, CDF19, CD_GE and Vehicle during 14 days (*n* = 7 in each group). *p < 0.05 compared to Vehicle-treated group (control) in respective day; the color of asterisk represents the comparing group

Body weight changes reflected the CDs toxicity, and this parameter’s decrease was strongly related to gross toxicity manifestations if any. Thus, there were tends down in body weight curve dynamics in CD_3011- and CDN19-treated groups, which were corroborated with mortality and signs of toxicity appearance (Fig. 8).

#### Biochemical assay

**Fig. 9:**
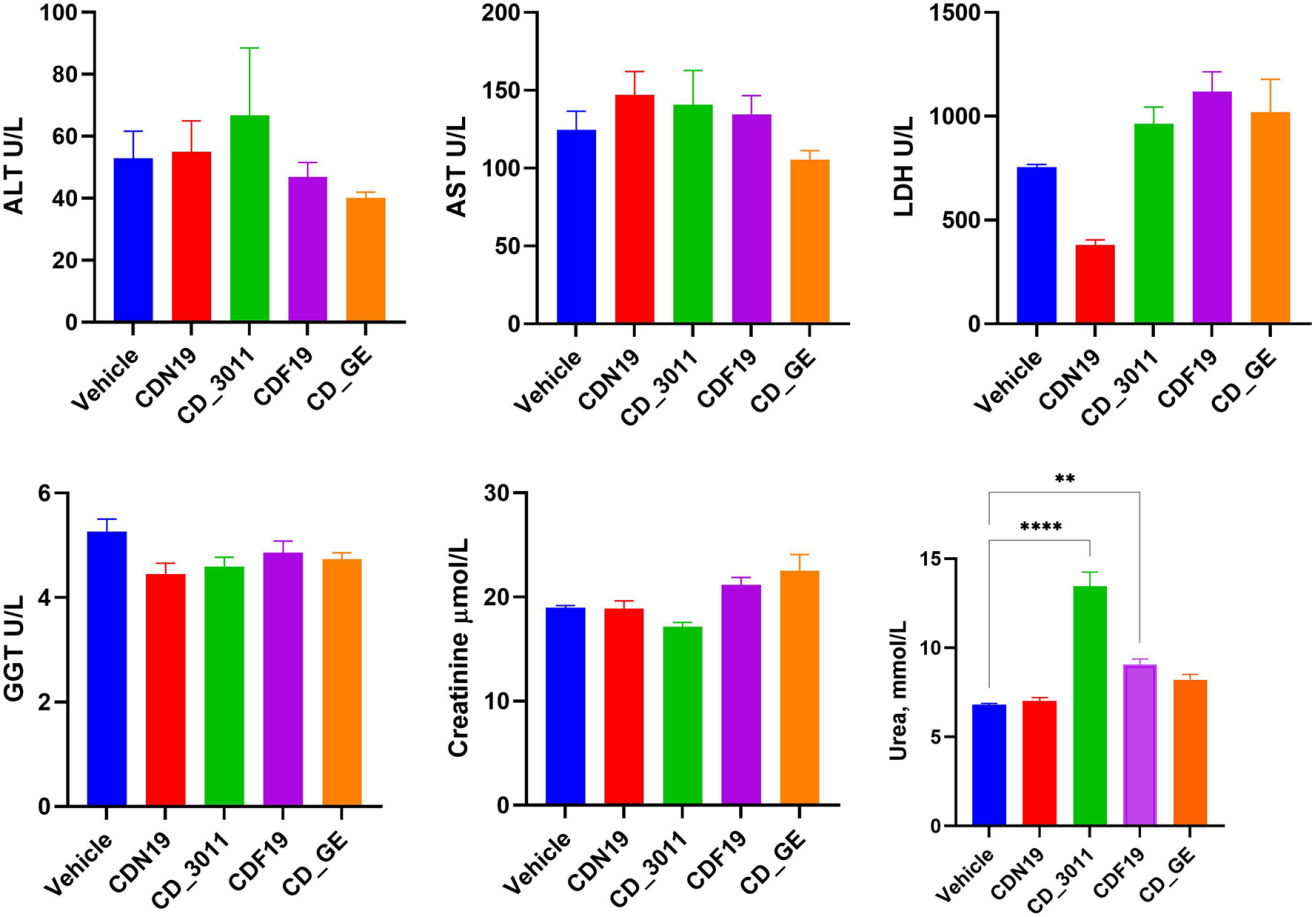
Serum biochemical parameters of mice treated with CDN19, CD_3011, CDF19, CD_GE and Vehicle (served as a control) during 14 days at the terminal day of the study (for survived mice: *n* = 3 for CDN19- and CD_3011-treated groups, *n* = 7 for CDF19-, CD_GE- and Vehicle-treated groups). **p < 0.01, ****p < 0.0001 compared to Vehicle

According to the data, there might be no substantial changes in serum biochemical markers; however, there were tends (significant or as a tend) to increase serum LDH activity and Urea content in CD_3011 and CDF19-treated mice (Fig. 9), which could indicate some alterations of liver and kidney caused by these CDs. There was also a substantial decrease in serum LDH activity in group treated with CDN19, which also could indicate the problems with liver. It should be noted, however, that in case of CDN19 and CD_3011, the data were obtained for survived mice only—obviously more tolerable for these CDs.

#### Microscopy assay of tissue samples

**Fig. 10:**
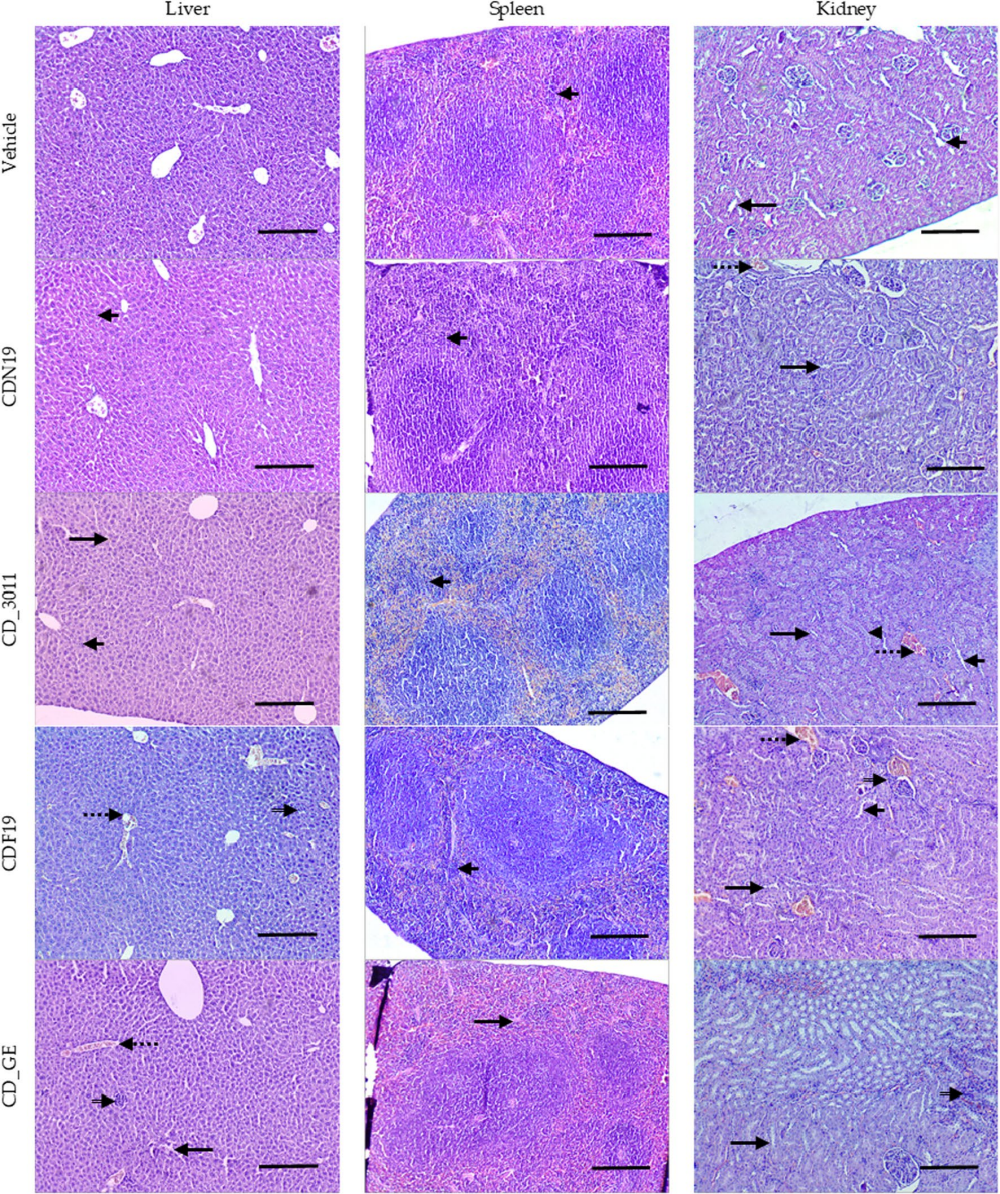
Representative microphotographs of kidney, liver and spleen of mice treated with CDs or a Vehicle (served as a control); liver: short arrows—Kupffer cell diffuse accumulation, long arrows—blood sinusoids dilation, empty arrows—lympho-histiocytes accumulation loci, dotted arrows—vessel congestion; spleen: short arrows—marginal zone hyperplasia, long arrow—connective tissue (fibrosis); kidneys: short arrows—tubular epithelium flattening, long arrows—loss of brush borders, arrowhead—epithelium vacuolation, empty arrows—interstitial nephritis, dotted arrow—vessel dilation. Magnification × 100, H&E. Scale 100 µm

According to histopathological data, tested CDs did not cause substantial injury of kidney, liver and spleen. The most impactful for kidneys was CD_3011, which caused slight injury of tubules manifested by epithelium flattening, sometimes epithelium desquamation (loss), loss of brush border, dystrophic changes (epithelium vacuolation), and some inflammatory reactions manifested by lympho-histiocytes accumulation surrounding glomerulus (glomerulonephritis) and blood vessel dilation, which could evidence an increased kidney blood supply. CDF19 caused similar but less pronounced changes (Table 5, Fig. 10).

**Table 5:** Pathological changes of kidney, liver and spleen which were observed in mice received CDs

	Vehicle	CDN19	CD_3011	CDF19	CD_GE
Kidney					
Tubular epithelium flattening	+	−	+	+	−
Loss of brush border	+	+	+	+	+
Tubular epithelium loss	−	−	+	−	−
Tubular epithelium vacuolation	−	−	++	−	−
Interstitial nephritis	−	−	−	+	+
Glomerulonephritis	−	−	+	−	−
Vessel dilation	−	+	++	++	−
Liver					
Hepatocellular hypertrophy	−	−	−	+	+
Vessel congestion	−	−	−	+	+
Lympho-histiocytes accumulation loci	−	−	−	+	+
Kupffer cell accumulation diffuse	−	+	+	−	−
Blood sinusoids dilation	−	−	++	−	+
Spleen					
Fibrosis (connective tissue accumulation)	−	−	−	−	+
Marginal zone hyperplasia	+	+	++	++	−

Trait intensity: “−”—not observed, “+”: single or slight, “++”: moderate

About liver—the most changes were caused by CD_GE, and they included hepatocellular hypertrophy, which might indicate liver function overload, and some alteration of blood supply manifested by vessel congestion and blood sinusoids dilation. Increased Kupffer cell diffuse accumulation in this group could be the consequence of CDs accumulation in liver and therefore tissue phagocytic cell attraction. CDF19 demonstrated similar but less pronounced consequences of its impact on liver (Table 5, Fig. 10).

Histopathological changes in spleen were restricted by marginal zone hyperplasia (from slight to moderate) throughout the groups (Table 5, Fig. 10), which might evidence some activation of phagocytic system because of being populated by macrophages predominantly [27]. Our data about potential CDs accumulation in spleen is in agreement with the literature [28, 29].

Thus, CDs CD_3011 and CDF19 probably affected kidney function, which is evidenced by serum biochemistry and histopathological data. CDs CD_GE and CDF19 could also affect liver as evidenced by liver pathohistological data, however, without significant damage of liver function (according to blood serum enzymes activity). This could be explained by liver high adaptive capacity—this organ could release its function even being significantly altered [30, 31]. It should be noted that serum ALT, AST, LDH, GGT, Creatinine and Urea levels of Vehicle-dosed mice were within the normal range, typical for this strain [32, 33].

## Discussion

According to our data, all tested CDs had no in vitro toxicity, however, demonstrated some in vivo after multiple dosing. Moreover, different CDs could reveal as immediate toxic response (after 2–3 days of exposure), as delayed one, which confirms the necessity of in vivo testing of the compounds especially if multiple dosing is expected.

As we have seen, the CDs toxicity differs depending on the surface modification. Despite no in vitro toxicity being observed for any of tested CDs, some was detected in vivo. Thus, CD_3011 (surface enriched with carboxyl groups) demonstrated the most overall and kidney-specific toxicity. Moreover, this CD had delayed effects probably because of accumulation, as evidenced by gross toxicity signs, mortality and body weight dynamics. On the contrary, CDN19 (surface enriched also with nitric groups) had the most expressed toxicity during the first days of administration (as evidenced by body weight dynamics and mortality). Then, the mice probably adapted and recovered, and there were no observable evidences of toxicity (histopathology, biochemistry) at the terminal day of the study. At any rate, toxicity of these CDs (CD_3011 and CDN19) after multiple dosing should be thoroughly considered if it will be decided to use these CDs as a background for drug design.

CDs are considered as low-toxic and quite biocompatible nanoparticles. For example, graphene quantum dots, which have similar structure to the tested CDs, demonstrated low or no toxicity against living organisms regardless to surface modification; however, they used single administration [14]. CDs if obtained from very different biological sources demonstrated very low in vitro toxicity so far [34].

Under physiological conditions, the core of CDs is supposed not to ionize into toxic species, have low to negligible undesirable reactivity, and rarely generate prolonged or significant inflammatory responses [35]. For example, Hong et al. [36] demonstrated that at doses up to 24 mg/kg administered every other day due to 1 months, there were no substantial evidence of toxicity in mice. Another study showed acute inflammatory response development after 1 week of CDs daily administrations but in the doses exceeded that in the current manuscript in 5 times [37].

According to the literature, CDs toxicity is related not to the exact surface modification, but to the surface charge, and the role of chemical groups on the surface restricted by the given charge. Thus, neutral or negatively charged CDs are the less toxic; however, negatively charged ones could induce oxidative stress. Whereas positively charged ones are the most cytotoxic [38, 39]. The main mechanism of cytotoxicity is considered to initialize oxidative stress, which leads to lipid peroxidation and therefore increase in membrane permeability [40] and other cellular events like G0/G1 and/or G2/M arrest [39]. In our in vitro studies, we demonstrated no toxicity for any CDs, which have the surfaces functionalized with oxygen-containing groups predominantly, so negatively charged, which is in line with the literature data [4, 39, 41].

Despite there were lots of studies dedicated to CDs in vitro toxicity, however, information regarding in vivo studies, especially those required multiple administrations are rare. Thus, acute toxicity of nitrogen-doped CDs was investigated in mice in doses similar to that we used and revealed no toxicity after single administration [42]. Another study demonstrated no obvious toxicity of CDs after prolonged administration every other day, however, immune system stimulation and impact on normal liver clearance (which is in agreement with our liver and spleen data) were shown [36]. In vivo multi-dosing toxicity test of graphene quantum dots showed no obvious toxicity of relatively safe polyethylene-glycolated ones, but animals’ mortality [43]. Thus, even if CDs revealed low or no toxicity after single-dose administration, some toxicity cannot be excluded after multiple dose administration.

As everyone proposes to use CDs for theranostics [9, 44 and elsewhere], so not only for bioimaging, i.e., diagnostics, which could require single administration, but for therapeutics, which obviously requires multiple dosages—the studies of CDs toxicity under sub-chronic and chronic administrations are quite necessary. They could uncover some adverse and/or delayed effects (as we observed in the present study) and to reveal much more information about CDs’ impact on living organisms.

Thus, CDs with different chemical compositions have different impact on mice after multiple everyday dosing, being as safe as harmful up to death depending on their structure. Some toxicity could appear during the first days of administration with subsequent recovery, or cumulative effects could occur as well. This should be taken into account during CDs selection for further studies and determining their dosing and treatment regimens. In other words, if the compound is safe after single-dose administration but toxic after multiple dosing—it does not necessarily mean that it is useless in the context of clinical applications. It might still be considered as an agent suitable for single dosage only, for instance, in bioimaging [9, 11]. However, caution must be exercised for any application that requires multiple administrations. These data are the background of further efficacy studies dedicated to discovering the potential antitumor activity of CDs.

### Supplementary Information

Additional file1 (DOCX 199 KB)

## Data Availability

All data generated and analyzed during the in vivo studies are included in this paper and its supplementary information. The datasets corresponding to the characterization of the CNs are available from the corresponding author on reasonable request.
