# Supplementary material for: In vitro and in vivo toxicity of carbon dots with different chemical compositions
Source: Discov Nano. 2023 Sep 8;18(1):111. doi: 10.1186/s11671-023-03891-9 (PMC10491573; doi:10.1186/s11671-023-03891-9)
Supplement: Supplementary file 1 — Additional file1 (DOCX 199 KB) [file 11671_2023_3891_MOESM1_ESM.docx]

**Supplementary materials**

*In vitro* and *in vivo* toxicity of carbon dots with different chemical compositions

Halyna Kuznietsova^1,2^*, Alain Géloën^3^, Natalia Dziubenko^1,2^, Alexander Zaderko^1,2^, Sergei Alekseev^4^, Vladimir Lysenko^5^ and Valeriy Skryshevsky^1,2^

^1^ Corporation Science Park, Taras Shevchenko University of Kyiv, 60 Volodymyrska Str., Kyiv, 01033, Ukraine

^2^ Institute of High Technologies, Taras Shevchenko National University of Kyiv, Volodymyrska Street, 64, 01601 Kyiv, Ukraine

^3^ Laboratoire Ecologie Microbienne (LEM), UMR CNRS 5557, INRAE 1418, VetAgroSup, Université Lyon 1, Domaine Scientifique de La Doua, 69100, Villeurbanne, France

^4^ Faculty of Chemistry, Taras Shevchenko National University of Kyiv, Lva Tolstoho Street, 12, 01033 Kyiv, Ukraine

^5^ Light Matter Institute, UMR-5306, Claude Bernard University of Lyon/CNRS, Université de Lyon, 69622, Villeurbanne Cedex, France

***** Correspondence: biophyz@gmail.com; Tel.: +380952774370


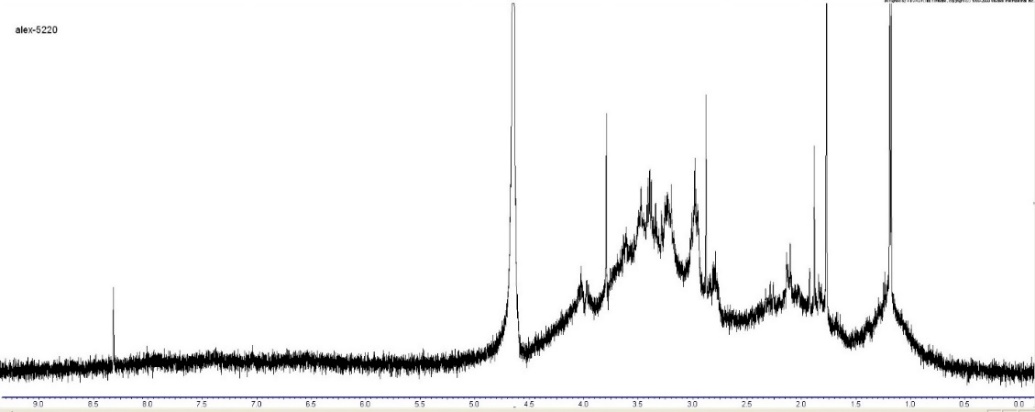


**Figure S1**. ^1^H NMR spectrum of CD_GE in D_2_O.


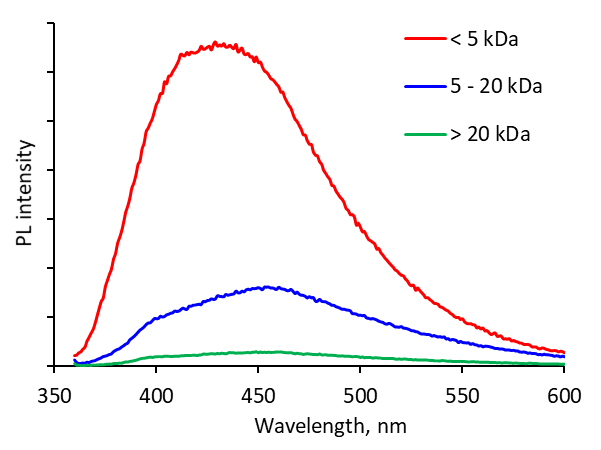


**Figure S2.** The PL spectra (λ_ex_ = 350 nm) of the CD_GE fractions (20x-diluted solutions).


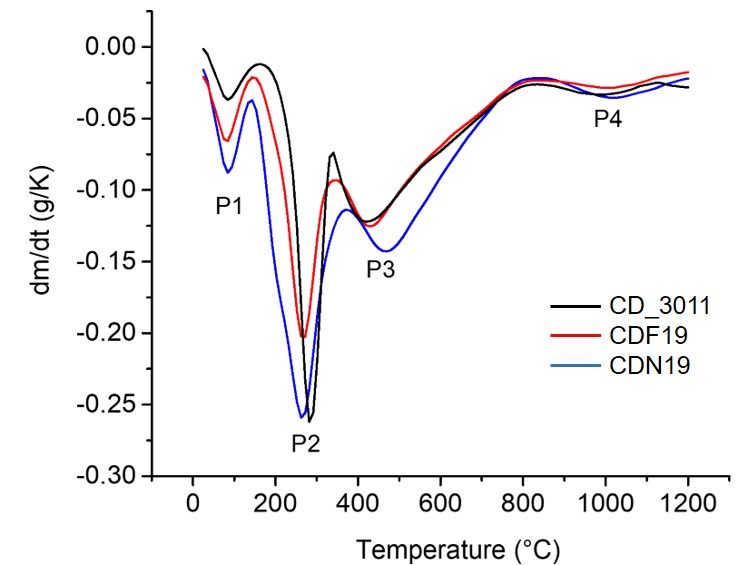


**Figure S3.** Differential thermogravimetry data for CD_3011, CDN19 and CDF19. Thermogravimetry peaks corresponds to weight loss rate depending on involving reactions: P1 - weight loss for physically sorbed water and other low molecular substances, e. g. alcohol added during the sample synthesis; P2 - chemical desorption of water during interaction of carboxyl groups with phenolic and amine ones; P3 - decarboxylation of carboxylic groups with CO_2_ release; P4 - weight loss from decomposition of quinone groups with partial carbon matrix destruction and CO release.

**Table S1.** Differential thermogravimetry peaks data for CD_3011, CDN19 and CDF19

| Sample |  | Weight loss peak, °C | | |
| --- | --- | --- | --- | --- |
|  | **Peak 1** | **Peak 2** | **Peak 3** | **Peak 4** |
| CD_3011 | 86.0 | 283.7 | 417.4 | 989.5 |
| CDF19 | 80.7 | 267.7 | 430.8 | 1007.0 |
| CDN19 | 83.2 | 262.3 | 466.9 | 1020.4 |

**
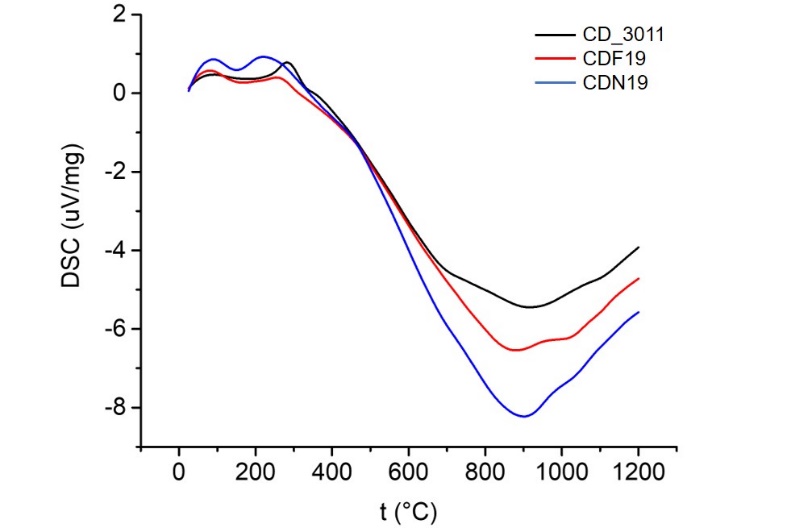
**

**Figure S4**. Differential scanning calorimetry for CD_3011, CDN19 and CDF19. Up to 250°C - insignificant effects due to the loss of sorbed water and low molecular weight substances; 250-360°C - dehydration (in fact, condensation) of surface functional groups; 360-900°C - functional groups decomposition, which affects the matrix (and is associated with CO desorption); 900-1200°C - moderate exothermic effect, apparently associated with the restoration of bonds in the carbon matrix due to the formation of polyconjugated and aromatic systems (partial graphitization).
